# Pre-existing immunity against vaccine vectors – friend or foe?

**DOI:** 10.1099/mic.0.049601-0

**Published:** 2013-01

**Authors:** Manvendra Saxena, Thi Thu Hao Van, Fiona J. Baird, Peter J. Coloe, Peter M. Smooker

**Affiliations:** 1 Ludwig Institute for Cancer Research, Heidelberg, Victoria, Australia; 2 School of Applied Sciences, RMIT University, Bundoora, Victoria, Australia; 3 Comparative Genomics Centre, School of Pharmacy and Molecular Sciences, James Cook University, Townsville, Queensland, Australia

## Abstract

Over the last century, the successful attenuation of multiple bacterial and viral pathogens has led to an effective, robust and safe form of vaccination. Recently, these vaccines have been evaluated as delivery vectors for heterologous antigens, as a means of simultaneous vaccination against two pathogens. The general consensus from published studies is that these vaccine vectors have the potential to be both safe and efficacious. However, some of the commonly employed vectors, for example *Salmonella* and adenovirus, often have pre-existing immune responses in the host and this has the potential to modify the subsequent immune response to a vectored antigen. This review examines the literature on this topic, and concludes that for bacterial vectors there can in fact, in some cases, be an enhancement in immunogenicity, typically humoral, while for viral vectors pre-existing immunity is a hindrance for subsequent induction of cell-mediated responses.

## Introduction

In the fields of medicine and veterinary medicine, there are numerous live, attenuated bacterial and viral vaccines in use today worldwide. The safety and efficacy of such vaccines is well established and allows further development as vector systems to deliver antigen originating from other pathogens. Various attenuated bacteria, including *Escherichia coli*, *Vibrio cholerae*, lactic acid bacteria (LAB), specifically *Lactococcus lactis*, *Mycobacterium*, *Listeria*, *Shigella* and *Salmonella*, have been tested for the targeted delivery of heterologous antigens of bacterial, viral and parasitic origin into a variety of animal hosts (Bahey-El-Din *et al.*, 2010; Innocentin *et al.*, 2009; Johnson *et al.*, 2011; Tobias *et al.*, 2008, 2010; Tobias & Svennerholm, 2012). Bacteria such as *E. coli* and lactic acid bacteria have recently gained favour, as *E. coli* is a commensal and lactic acid bacteria are present in most fermented food items and are therefore naturally present in the host. They are also a much safer option than traditional attenuated vaccines in children and immune-compromised people. As this review discusses the effects of pre-existing immune responses to attenuated vaccines, further discussion of LAB and *E. coli* as potential vectors will not be undertaken; however, the reader is directed to several interesting reviews (Bermúdez-Humarán *et al.*, 2011; Wells & Mercenier, 2008).

**Fig. 1.**
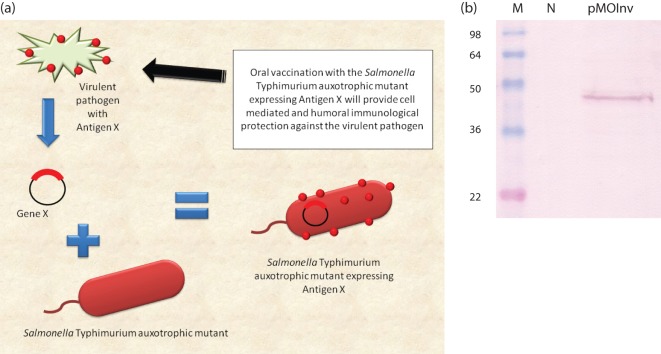
(a) General approach to using bacteria as vaccine vectors. In this case, the heterologous antigen is depicted as being expressed on the bacterial surface. (b) *Salmonella* secretion of heterologous antigen. STM-1 was engineered to secrete a haemolysin protein. Western blot probed with anti-haemolysin antisera. M, Marker lane (in kDa); N, concentrated growth medium after STM-1 growth (no plasmid); pMOInv, medium after growth of STM-1 with the pMOInv plasmid, encoding haemolysin.

Intracellular bacteria from the genera *Mycobacterium* (Guleria *et al.*, 1996), *Listeria* (Gentschev *et al.*, 2001), *Shigella* (Levine *et al.*, 1997) and *Salmonella* (Dougan *et al.*, 1987) are considered to be suitable candidates for the delivery of vaccine antigens due to their capability to induce robust T cell immune responses (Alderton *et al.*, 1991; Lo *et al.*, 1999; Mastroeni *et al.*, 2001; Mittrücker & Kaufmann, 2000; Nauciel, 1990). *Salmonella* is one genus that has been well examined as a vector, building on the extensive research available on the micro-organism’s physiology and pathogenesis (Basso *et al.*, 2000; Killeen & DiRita, 2000; Sirard *et al.*, 1999; Ward *et al.*, 1999). There exist several commercial vaccines that are used as anti-*Salmonella* vaccines in humans and animals (e.g. Ty21a for typhoid fever in humans, several *Salmonella* serovars against salmonellosis in chickens and other animals). The general strategy for vectoring heterologous antigen is depicted in Fig. 1. The first clinical trial of a recombinant, which was conducted over 20 years ago using an attenuated *Salmonella* as a delivery vector, led to the widespread testing of this bacterium as a mucosal delivery system for antigens from non-*Salmonella* pathogens (Dougan *et al.*, 1987). These studies have demonstrated the utility of live bacteria to deliver expressed antigens and DNA vaccines to the host immune system (Atkins *et al.*, 2006; Husseiny & Hensel, 2008; Jiang *et al.*, 2004; Kirby *et al.*, 2004). Since then several other intracellular bacterial vectors have been successfully tested for their capability to deliver a variety of antigens from various pathogens, as well as vaccination against cancer. One genus which has been widely tested as vector is *Listeria*. *Listeria* species are Gram-positive intracellular food-borne pathogens. The advantages of *Listeria* are that it can invade a variety of cells, including antigen presenting cells (APCs). After invading the host cell, *Listeria* resides inside the phagosome; however, it can escape the phagosome with the help of listeriolysin O (LLO; Hly) and reside in the cytoplasm of the cells, thereby efficiently presenting antigen to both CD8 and CD4 T cells (Cossart & Mengaud, 1989; Kaufmann, 1993; Pamer *et al.*, 1997). Several studies have demonstrated the effectiveness and ease of using *Listeria monocytogenes* to deliver heterologous vaccine antigens and DNA vaccines (Brockstedt *et al.*, 2004; Jensen *et al.*, 1997; Johnson *et al.*, 2011; Peters *et al.*, 2003; Shen *et al.*, 1995; Yin *et al.*, 2011).

Similarly, various viral vectors have been successfully tested for their capability to deliver heterologous vaccine antigens, and this generally results in the induction of strong CTL immune responses. In the veterinary field, there are numerous viral vector vaccines that are currently licensed for use in livestock and domesticated animals. These recombinant vaccines are based on both DNA viruses (such as fowlpox virus-based vaccines which target avian influenza virus and fowlpox virus, or vaccinia virus-based vectors against the rabies virus in wildlife) and RNA viruses [such as Newcastle disease virus-based vaccines to be used in poultry or yellow fever virus (YFV)-based vaccines to be used in horses against West Nile virus] (Draper & Heeney, 2010). Based on the safety record in the veterinary field, many viruses have been studied for human use as a vector in vaccine development (Beukema *et al.*, 2006; Esteban, 2009; Schirrmacher & Fournier, 2009; Stoyanov *et al.*, 2010; Weli & Tryland, 2011). Amongst them, YFV (YF-17D strain) was the first to be licensed for use in humans, where the cDNAs encoding the envelope proteins of YFV were replaced with the corresponding genes of an attenuated Japanese encephalitis virus strain, SA14-14-2 (Appaiahgari & Vrati, 2010; Rollier *et al.*, 2011). Poxviruses are also studied extensively as candidate vectors for human use, among which attenuated derivatives of vaccinia virus [such as modified vaccinia virus Ankara (MVA) and New York attenuated vaccinia virus NYVAC strains] are the most promising vectors (Esteban, 2009; Gómez *et al.*, 2008; Rimmelzwaan & Sutter, 2009). They are ideal candidate vectors due to their large DNA-packing capacity and their thermal and genetic stability (Minke *et al.*, 2004). The NYVAC vector has been shown to induce CD4^+^ T cell-dominant responses, and MVA induces both CD4^+^ and CD8^+^ T cell responses (Mooij *et al.*, 2008). The adenovirus (Ad) vector is another of the most widely evaluated vectors to date to express heterologous antigens, due to ease of production, safety profile, genetic stability, the ease of DNA genome manipulation, and the ability to stimulate both innate and adaptive immune responses and induce both T and B cell responses (Alexander *et al.*, 2012; Fitzgerald *et al.*, 2003; Gabitzsch & Jones, 2011; Lasaro & Ertl, 2009; Vemula & Mittal, 2010; Weyer *et al.*, 2009). They have been extensively examined as a delivery vector in several preclinical and clinical studies for infectious diseases such as anthrax, hepatitis B, human immunodeficiency virus (HIV)-1, influenza, measles, severe acute respiratory syndrome (SARS), malaria and tuberculosis (Chengalvala *et al.*, 1994; Gao *et al.*, 2006; Hashimoto *et al.*, 2005; Hsu *et al.*, 1992; Limbach & Richie, 2009; Radosevic *et al.*, 2007; Shiver *et al.*, 2002).

However, before vectored vaccines can be used in the human population they need to satisfy several important criteria. Safety is a major concern, as even a low level of toxicity is unacceptable (of course the minor discomfort that accompanies many vaccinations is normal). Secondly, a vaccine should be inexpensive, so that it can be administered to a large population at minimal cost, and this is particularly important in resource-poor countries (Killeen & DiRita, 2000). Similar constraints apply to veterinary vaccines, with cost often an even more important consideration. Finally, long-lasting cellular and (where appropriate) humoral immune responses to the vectored antigen must be induced following administration of these vaccines, preferably with a single dose (Atkins *et al.*, 2006).

As some of the vectors in use will have been seen by the host immune system prior to vaccination, whether the presence of pre-existing immune responses is detrimental for the further development of a vector-based vaccine scheme, or can augment responses to the vectored antigen, needs to be considered in detail. This is the subject of this review. In discussing the possible effects on pre-existing immunity, the natural immunity to the vector needs to be considered. Therefore, considering a vector such as *Salmonella*, if a host has previously been infected there will exist robust B and T memory responses, and as such, when a vaccination is delivered, an anamnestic response to the *Salmonella* antigens will be induced (while the response to the vectored antigen will be a primary response). This will theoretically reduce the exposure of the heterologous antigen to the immune system, as the vector is rapidly cleared. Surprisingly, as will be seen in some of the examples given below, this can have results that differ depending on the magnitude of the response to the vectored antigen. Similarly, for virally vectored antigens, the existence of pre-existing immunity to the vector (particularly neutralizing antibody) will restrict delivery of the virus into cells, thereby effectively reducing the dose of the vectored antigen. Again, this might be expected to result in a reduction in the antigenicity of the vectored antigen.

## Effects of prior immunological exposure to vectors – bacterial vectors

In the case of bacterial vectors, the effect of pre-existing immune responses has only been tested using *Salmonella* serovars and *Listeria* spp. Concern that prior immunological experience of the host with either the homologous *Salmonella* vector strain or a related strain might compromise its ability to deliver heterologous vaccine antigen was first raised in 1987 (Dougan *et al.*, 1987). Bao and Clements subsequently reported experimental evidence of the consequences of prior exposure of animals to the vector strain (Bao & Clements, 1991). This work showed that both serum and mucosal antibody responses against the foreign antigen were in fact upregulated in animals with prior exposure to the vector strain. Whittle & Verma (1997) reported similar findings. Mice immunized via the intra-peritoneal route with a *Salmonella dublin aroA* mutant expressing heterologous antigen after being exposed to the same vector showed a higher immune response to the vectored antigen in comparison to mice without any immunological memory against the vector.

Subsequently, several studies have been conducted to examine the effect of pre-existing immunity in the host against *Salmonella.* These results are summarized in Table 1. The various reports are contradictory in their findings and seem to paint a rather confusing picture. Some studies concluded that pre-existing immunity against the *Salmonella* vector leads to stronger immune responses against the delivered antigen (Bao & Clements, 1991; Jespersgaard *et al.*, 2001; Kohler *et al.*, 2000a, b; Metzger *et al.*, 2004; Saxena *et al.*, 2009; Sevil Domènech *et al.*, 2008; Whittle & Verma, 1997), with others considering pre-existing immunity to be a limiting factor in the long-term use of *Salmonella* as an efficient vector for antigen delivery (Attridge *et al.*, 1997; Gahan *et al.*, 2008; Roberts *et al.*, 1999; Sevil Domènech *et al.*, 2007; Vindurampulle & Attridge, 2003a, b).

**Table 1.** Summary of published reports and their conclusions na, Not applicable; nd, not determined.

Vaccine recipient	Vaccine vector	Pre-existing immunity organism	Vectored antigen	CMI response	Humoral response	Reference
Mouse	*S.* *dublin*	*S. dublin*	na	nd	+	Bao & Clements (1991)
Mouse	*S. dublin*	*S. typhimurium*	na	nd	++	Bao & Clements (1991)
Mouse	*S. typhimurium*	*S. typhimurium*	Glucan-binding domain of glucosyltransferase, *Streptococcus mutans*	nd	++	Jespersgaard *et al.* (2001)
Mouse	*S. typhimurium*	*S. typhimurium*	Haemagglutinin, *Porphyromonas gingivalis*	nd	++	Kohler *et al.* (2000a, b)
Human	*S. typhi* Ty21a	*S typhi* Ty21a	Urease subunits A and B, *Helicobacter pylori*	No change	++	Metzger *et al.* (2004)
Mouse	*S. typhimurium*	*S. typhimurium*	Ovalbumin, *G. gallus*	++	+++	Saxena *et al.* (2009)
Mouse	*S. typhimurium*	*Salmonella enterica* serovar Enteritidis	Ovalbumin, *G. gallus*	++	++	Saxena *et al.* (2009)
Mouse	*S. dublin*	*S. typhimurium*	Fusion protein of *Yersinia* outer protein E and p60 from *L. monocytogenes*	++	nd	Sevil Domènech *et al.* (2008)
Mouse	*S. typhimurium*	*S. dublin*	Fusion protein of *Yersinia* outer protein E and p60 from *L. monocytogenes*	++	nd	Sevil Domènech *et al.* (2008)
Mouse	*S. dublin*	*S. dublin*	Envelope protein, Murray Valley encephalitis virus	nd	+++	Whittle & Verma (1997)
Mouse	*S. stanley*	*S. stanley*	Fimbrial protein K88, *E. coli*	nd	−−−	Attridge *et al.* (1997)
Mouse	*S. stanley*	*Salmonella strasbourg*	Fimbrial protein K88, *E. coli*	nd	−	Attridge *et al.* (1997)
Mouse	*S. typhimurium*	*S. typhimurium*	C fragment of tetanus toxin, *Clostridium tetani*	nd	−−	Gahan *et al.* (2008)
Mouse	*S. typhimurium*	*S. typhimurium*	C fragment of tetanus toxin, *C. tetani*	nd	−−−	Roberts *et al.* (1999)
Mouse	*S. typhimurium*	*S. dublin*	C fragment of tetanus toxin, *C. tetani*	nd	−−	Roberts *et al.* (1999)
Mouse	*S. typhimurium*	*S. typhimurium*	Fusion protein of *Yersinia* outer protein E and p60 from *L. monocytogenes*	−−−	nd	Sevil Domènech *et al.* (2007)
Mouse	*S. stanley*	*S. stanley*	Fimbrial protein K88, *E. coli*	nd	−−−	Vindurampulle & Attridge (2003b)
Mouse	*S. dublin*	*S. stanley*	Fimbrial protein K88, *E. coli*	nd	−−−	Vindurampulle & Attridge (2003b)

**Fig. 2.**
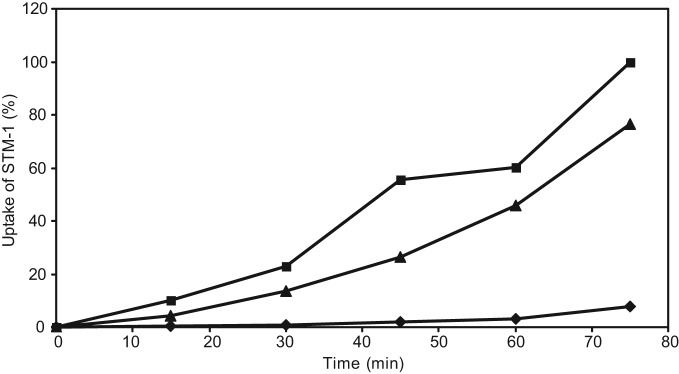
Uptake of STM-1 by J774 macrophages, relative to the highest uptake percentage. ⧫, Opsonized with naive sera; ▴, opsonized with serum from mice exposed to *Salmonella enteriditis*; ▪, opsonized with serum from mice exposed to STM-1.

A slight majority of the studies listed in Table 1 (10 versus eight) indicate the upregulation of immune responses after animals have been exposed to either homologous or related strains before the delivery of heterologous antigen using a *Salmonella* vector. A study by Metzger and co-workers on human volunteers using *Salmonella* Typhi as a vector suggested that there was no change in the T cell immune response against the heterologous antigen in human volunteers who were exposed to empty vector in comparison with volunteers who were immunologically naive of the vector strain (Metzger *et al.*, 2004). In these subjects, humoral responses were moderately elevated in pre-exposed individuals. Similarly, Saxena *et al.* (2009) indicated higher humoral and T cell responses in mice pre-exposed to homologous or heterologous *Salmonella* strains. The interleukin 4 (IL4) response was significantly higher when the animal host was exposed to the homologous strain, whereas pre-exposure to a related species did not have such an impact on IL4 responses. Conversely interferon (IFN)-γ responses were higher, irrespective of the strain to which mice were pre-exposed. This study also indicated that the presence of homologous or heterologous opsonizing antibodies leads to a higher uptake of *Salmonella* by macrophages *in vitro*, which may explain the higher immune responses in exposed mice. As may be expected, uptake was higher when homologous sera were used as the opsonin rather than heterologous sera. This is depicted in Fig. 2.

Conversely, there are reports that indicate that pre-existing immunity against the bacterial vector downregulates immune responses against the delivered heterologous antigen using similar or related vectors. Attridge and co-workers reported that the presence of immunity against the bacterial vector prior to the delivery of vectored antigenic protein can downregulate immune responses in mice against the delivered antigen (Attridge *et al.*, 1997). Similar results were reported by Roberts *et al.* (1999) and Vindurampulle & Attridge (2003a, b). However, the latter authors found that the hypo-responsiveness could be largely eliminated by exposing animals to the foreign antigen prior to vector-priming (Vindurampulle & Attridge, 2003b). Unfortunately, this would appear to be impractical for an immunization regimen!

A study presented by Gahan *et al.* (2008) immunized mice with *S.* Typhimurium expressing C fragment of tetanus toxin antigen from an expression plasmid or as a DNA vaccine. Vaccinated mice developed humoral responses to LPS and tetC (for the plasmid-bearing vaccines). Animals from all groups (including a previously unvaccinated group) were immunized on day 182 with *Salmonella* expressing tetC. At this time, the anti-LPS and tetC titres were beginning to wane. Fourteen days after the second immunization, the colonization of various mouse organs was assessed. The ability to colonize was found to be significantly reduced in groups that had been previously vaccinated with *Salmonella*. In view of this finding, it was perhaps not surprising that at day 210 the LPS titres were not significantly different between groups receiving one or two vaccinations. More interestingly, mice that had been primed with *Salmonella* alone, and then boosted with *Salmonella* expressing tetC, induced much lower anti-tetC responses than mice that had not been primed. This argues strongly that prior immunological immunity to the vector can seriously dampen subsequent antigen-specific humoral responses. Whether the same is true for cellular responses was not evaluated.

Other studies have evaluated cellular responses. A study by Sevil Domènech and colleagues reported that pre-existing anti-vector immunity seriously compromises CD8^+^ responses in mice when exposed to a similar strain used as vector (Sevil Domènech *et al.*, 2007). In contrast, another study by the same authors reported that animals exposed to related vectors induce much higher CD8^+^ responses when compared with animals which do not have any pre-existing *Salmonella* immunity (Sevil Domènech *et al.*, 2008). The difference between these two studies was that in the first, the prime and boost were with identical serovars, while in the second study, different serovars were used. This may point to a way of avoiding downregulation of CD8 responses by pre-existing immunity. This is important, as one of the advantages of using *Salmonella* (an intracellular pathogen) is that strong cellular immune responses can be induced.

It must be noted that in the case of *Salmonella* vaccines, effects other than strictly immunological responses (particularly adaptive responses) should be considered. In the context of innate immunity, it was shown that administration of non-virulent *Salmonella* to gnobiotic pigs eliminated disease following challenge with a virulent strain (Foster *et al.*, 2003). Interestingly, protection was not by competitive exclusion, as the virulent strain was in high numbers in the gut but did not distribute systemically. The protection was proposed to be mediated by the infiltration of a large number of polymorphonuclear leukocytes into the gut, and although perhaps impractical as a general prophylactic (as the time between vaccination and infection is short), this may be an option for short-term or perhaps therapeutic vaccination (as reviewed by Foster *et al.*, 2012).

Chickens (*Gallus gallus*) are a natural animal reservoir for *Salmonella*, which makes them an important source of *Salmonella*-associated gastroenteritis in humans. The ability to use oral *Salmonella* vaccines to immunize against heterologous pathogens would be of enormous benefit to the poultry industry in both broiler and layer flocks. Both vertical and horizontal transmission is associated with *Salmonella* in chickens (Liljebjelke *et al.*, 2005). Vertical transmission via *in ovo* transmission is particularly important, because if there is prior exposure to the vaccine strain, subsequent vaccination using an oral *Salmonella* vector could be severely compromised. A considerable number of studies on cross-protective immunity and competitive exclusion have been undertaken in chickens. Protective cross-reactive immunity against *Salmonella* strains has been demonstrated against both homologous and heterologous challenges (Beal *et al.*, 2006), although cross-serogroup protection was not strong. Furthermore, a recent study reported that pre-treatment of newly hatched chickens with different *Salmonella* strains could produce a complete invasion–inhibition effect on any subsequent exposure to both homologous and heterologous strains (Methner *et al.*, 2010). Pre-exposure with a highly invasive form of *Salmonella* Enteritidis caused a large influx of heterophils to the caecal mucosa in 1-day-old chicks, and subsequent heterologous caecal colonization was inhibited for a period of 48 h (Methner *et al.*, 2010). The implications of this kind of colonization-inhibition study on the immunological status of the affected chickens are yet to be fully elucidated. It should be noted that the studies listed in Tables 1 and 2 are controlled laboratory studies, with the possibility of a competitive exclusion component to immunity not discussed.

**Table 2.** Different effects of pre-existing immunity on the efficacy of recombinant viral vaccine vectors nd, Not determined.

Vaccine recipient	Vaccine vector	Pre-existing immunity organism	Vectored antigen	CMI response	Humoral response	Reference
Mouse	Poliovirus	Poliovirus	Chicken ovabumin	−	No change	Mandl *et al.* (2001)
Mouse	HSV	HSV	Chicken ovabumin	−−−	−−−	Lauterbach *et al.* (2005)
Mouse	HSV	HSV	*E. coli* β-galactosidase	No change	No change	Brockman & Knipe (2002)
Mouse	Ad	Ad	HIV-1 gag	−−−	nd	Pichla-Gollon *et al.* (2009)
Human	Ad	Ad	HIV-1 gag/pol/nef	−−	nd	McElrath *et al.* (2008)
Mouse	Ad	Ad	H5 haemagglutinin	−	−	Alexander *et al.* (2012)
Mouse	Ad	Ad	Ovabumin/glycoprotein of lymphocytic choriomeningitis virus	−	nd	Steffensen *et al.* (2012)
Mouse	Ad	Ad	H5 haemagglutinin and N1 nucleoprotein	−−	−−	Pandey *et al.* (2012)

Similarly studies of *L. monocytogenes* and the effects of pre-existing immune responses indicate conflicting results. A study by Bouwer *et al.* (1999) indicates that pre-existing immune responses against the *Listeria* vector do not diminish immune responses against the delivered heterologous antigen, and a similar study by Starks *et al.* (2004) also concluded that prior exposure of mice to the empty *Listeria* vector did not influence anti-cancer immune responses when a similar mutant was used as a carrier of a melanoma cancer antigen. Similar findings were reported by Whitney *et al.* (2011) in rhesus macaques in which *L. monocytyogens* was used as a carrier of gag-HIV antigen. Conversely, studies by Stevens *et al.* (2005) in which *L. monocytogens* was used to deliver feline immunodeficiency virus (FIV) gag protein and as a carrier of DNA vaccines to vaccinate cats against FIV envelope protein indicated lower immune responses against the delivered antigen in cats exposed to empty *Listeria* vector in comparison with naive animals (Stevens *et al.*, 2005). Similar findings have been reported by Tvinnereim *et al.* (2002) and Leong *et al.* (2009). However, taken together, these studies conclude that prior exposure of host animals to empty vector does not abrogate immune responses to the vectored antigen, but only reduces them somewhat. Only the study by Vijh *et al.* (1999) indicated that exposure to the empty vector may completely abrogate immune responses against the delivered antigens (Vijh *et al.*, 1999). However, these studies also indicate that downregulation of antigen-specific immune responses is highly dependent on dose and time. Leong *et al.* (2009) also demonstrated that the negative impact of vector-specific immune responses can also be countered by repeated immunization with the same vaccine and dose; this in effect leads to higher priming of naive T cells against the delivered antigen. Of course, such repeated vaccination may not be practicable in real-world situations.

## Effects of prior immunological exposure to vectors – viral vectors

Despite the many advantages which viral vectoring can offer, pre-existing immunity is a major obstacle of many viral-vectored vaccines, such as Ad serotype 5 or herpes simplex virus type 1 (HSV-1), where the rate of seroprevalence to these viruses is very high [40–45 % and 70 % (or more) of the US population, respectively] (Hocknell *et al.*, 2002; Pichla-Gollon *et al.*, 2009). Vector-specific antibodies may impede the induction of immune responses to the vaccine-encoded antigens, as they may reduce the dose and time of exposure of the target cells to the vaccinated antigens (Pichla-Gollon *et al.*, 2009; Pine *et al.*, 2011). In a large-scale clinical trial (STEP) of an Ad serotype 5 (AdHu5)-based HIV-1 vaccine, the vaccines showed a lack of efficacy and tended to increase the risk of HIV-1 infection in vaccine recipients who had pre-existing neutralizing antibodies to AdHu5 (Buchbinder *et al.*, 2008). For an HSV-1-based vector vaccine, it has been demonstrated that pre-existing anti-HSV-1 immunity reduced, but did not abolish, humoral and cellular immune responses against the vaccine-encoded antigen (Hocknell *et al.*, 2002; Lauterbach *et al.*, 2005). However, Brockman and Knipe found that the induction of durable antibody responses and cellular proliferative responses to HSV-encoded antigen were not affected by prior HSV immunity (Brockman & Knipe, 2002). Similarly, pre-existing immunity to poliovirus has little effect on vaccine efficacy in a poliovirus-vectored vaccine (Mandl *et al.*, 2001). Different effects of pre-existing immunity on the efficacy of recombinant viral vaccine vectors are summarized in Table 2. There are several approaches to avoiding pre-existing vector immunity, such as the use of vectors derived from non-human sources, using human viruses of rare serotypes (Kahl *et al.*, 2010; Lasaro & Ertl, 2009), heterologous prime–boost approaches (Liu *et al.*, 2008), homologous reimmunization (Steffensen *et al.*, 2012) and removing key neutralizing epitopes on the surface of viral capsid proteins (Gabitzsch & Jones, 2011; Roberts *et al.*, 2006). The inhibitory effect of pre-existing immunity can also be avoided by masking the Ad vector inside dendritic cells (DCs) (Steffensen *et al.*, 2012). In addition, mucosal vaccination or administration of higher vaccine doses can overcome pre-existing immunity problems (Alexander *et al.*, 2012; Belyakov *et al.*, 1999; Priddy *et al.*, 2008; Xiang *et al.*, 2003).

## Concluding remarks and perspective

As we search for new vaccine approaches for the array of pathogens for which none is yet available, revisiting proven vaccines and developing these further has gained momentum. Hence, attenuated bacteria and viruses which have a long history of efficacy and safety are being brought into use. While very attractive, a common theme in these experimental approaches has been the limitations that pre-existing immunity to the vector may pose. However, as this examination of the relevant literature shows, there is a rather confusing picture, with some studies in fact indicating that pre-existing immunity may be a friend, rather than foe.

Few studies using viral vectors have reported on the influence of pre-existing immunity on humoral responses. Generally speaking, for bacterial-delivered antigens, the humoral responses were influenced by pre-existing immunity, with slightly more studies finding augmentation rather than diminution. Why is there variation? This may be due to several factors, including the type of *Salmonella* used and its invasiveness. Dunstan and colleagues tested the ability of six isogenic *Salmonella* serovar Typhimurium strains harbouring different mutations for their ability to induce immune responses against the C fragment of tetanus toxin and concluded that the strain which had the least ability to colonize Peyer’s patches induced the lowest immune responses (Dunstan *et al.*, 1998).

Similarly, the boosting time and nature of the antigen used might be important. Attridge and colleagues indicated the importance of boosting time. In one experiment, boosting mice at 10 weeks led to complete inhibition of antibody responses against the delivered heterologous antigen; however, when the mice were boosted at 4 weeks, the downregulation of antibody responses was not so prominent (Attridge *et al.*, 1997). A similar study conducted by Kohlers and colleagues shows that boosting at 7 weeks after pre-exposing animals to empty vector leads to lower antigen-specific IgG and secretory IgA responses; however, boosting at 14 weeks leads to higher IgG and secretory IgA responses (Kohler *et al.*, 2000b). This is in conflict with the above result, although it should be mentioned that they used different *Salmonella* species. Vindurampulle and Attridge also examined the impact of the *Salmonella* strain and the nature of the antigens used. In their study, they used *S.* Dublin and *Salmonella* Stanley *aroA* mutants to deliver *E. coli* K88 and LT-B antigens, and concluded that the effect of pre-existing immunity depends on both the strain used and the type of antigen delivered (Vindurampulle & Attridge, 2003b).

All these studies on the effect of pre-existing immunity discuss the impact on humoral responses. Sevil Domenech and colleagues reported that pre-exposing animals to the homologous *Salmonella* vector leads to a significant reduction in CD8^+^ responses; however, exposure of animals to a heterologous strain leads to significantly higher CD8^+^ responses (Sevil Domènech *et al.*, 2007, 2008). Saxena and colleagues also reported that antigen-specific T cell responses were either similar or significantly higher, with no downregulation in T cell responses observed after pre-exposing mice to either homologous or heterologous strains (Saxena *et al.*, 2009).

For viral vectors, the impact of cell-mediated immunity was more pronounced, and as depicted in Table 2, almost always resulted in a reduction in the subsequent immune response. Presumably this is because viruses will induce neutralizing antibody on the first dose, and in subsequent doses this antibody will limit the number of transduced cells, therefore limiting the responses. This is particularly a problem with a common viral vector such as Ad, where a large proportion of the population will have immunological memory against common serotypes (Lasaro & Ertl, 2009). As these authors conclude, it will be possible to utilize such vectors only by developing vaccines from alternative serotypes. It may be that a vector such as attenuated influenza virus, with the ability to easily develop reassortants, will be useful in this context.

In addition, immunological memory in the form of opsonizing antibody certainly plays an important role in the early uptake of *Salmonella* by macrophages and DC. This may be beneficial, as the live bacterial vector used for delivery purposes harbours mutations in genes encoding proteins responsible for their survival in the animal host. This not only encumbers their ability to cause disease, making them safe live vectors, but also limits the number of replications. The presence of opsonizing antibodies should mean a higher level of bacterial uptake, leading to higher presentation to the immune system and therefore a better immune response. We have previously shown that this is indeed the case (Saxena *et al.*, 2009) (depicted in Fig. 2). It would be of great benefit to address these issues not only in mice but also in other organisms such as chickens, which are the most likely host to be targeted for the use of live *Salmonella* vectors, specifically where the vaccines are developed for use in livestock and poultry.

To summarize, bacterial vectors such as *Salmonella* and viral vectors such as Ad show great promise as delivery vehicles for heterologous antigens; however, prior exposure to the vector must be considered. By judicious selection of the strain/serotype it will be possible to avoid the negative effects and it may indeed be possible to positively influence the response, particularly for humoral immunity.
